# Characteristics of Swallowing Function in People with Parkinson's Disease: A Scoping Review

**DOI:** 10.1002/mds.70164

**Published:** 2026-01-08

**Authors:** Kerstin Erfmann, Julia Hirschwald, Jule Hofacker, Katharina Winiker, Juliane Klann, Rainer Dziewas, Tobias Warnecke

**Affiliations:** ^1^ Faculty of Business Management and Social Sciences, Section of Speech and Language Therapy University of Applied Sciences Osnabrück Osnabrück Germany; ^2^ Department of Neurology Klinikum Osnabrück Osnabrück Germany; ^3^ Clinical Speech and Language Studies Trinity College Dublin Dublin Ireland; ^4^ Heinrich‐Heine‐University Düsseldorf Düsseldorf Germany; ^5^ Department Research and Development Swiss University of Speech and Language Sciences (hlo) St. Gallen Switzerland; ^6^ School of Health, Education and Social Sciences, Campus Heidelberg SRH University of Applied Sciences Heidelberg Heidelberg Germany

**Keywords:** dysphagia, Parkinson's disease, scoping review, speech‐language pathology, swallowing

## Abstract

**Background:**

Most individuals with Parkinson's disease (PD) develop dysphagia during the course of their disease. It is crucial to comprehensively understand swallowing characteristics specific to PD for effective treatment.

**Objectives:**

To systematically analyze and synthesize swallowing characteristics in people with PD compared with healthy controls and to summarize the assessment methods used.

**Methods:**

This scoping review was conducted in accordance with the Joanna Briggs Institute's (JBI) methodology. Systematic searches were conducted across six databases and one clinical trial registry. Original research articles reporting swallowing characteristics in cohorts comprising at least three individuals with PD were included. Studies published in English or German from the inception of the databases up to February 2024 were considered for inclusion. Independent reviewers assessed articles for eligibility and extracted relevant data. The scoping review protocol was registered and published (Open Science Framework, https://osf.io/8b3hm). Swallowing characteristics and assessment methods were systematically categorized. Significant characteristics, consistent across at least two studies, were included in the final analysis.

**Results:**

Analysis included 46 studies with 1797 PD participants aged 35–85 years. Nineteen distinct oropharyngeal swallowing characteristics in individuals with PD were identified. Instrumental assessments (39/46, 85%), clinical assessments (10/46, 22%), and patient‐related outcome measures (PROMs) (7/46, 15%) were used.

**Conclusions:**

Relevant swallowing abnormalities in PD can largely be interpreted as manifestations of bradykinesia, hypokinesia, and akinesia, supporting the view that dysphagia in PD constitutes a complex motor syndrome. Future research should aim to better characterize the manifestations within this syndrome and elucidate its underlying pathology. © 2026 The Author(s). *Movement Disorders* published by Wiley Periodicals LLC on behalf of International Parkinson and Movement Disorder Society.

Impaired swallowing, or dysphagia, is common in individuals with Parkinson's disease (PD) with prevalence rates reported as high as 81%, depending on the definition and assessment methods employed.^1^ Dysphagia can manifest at any disease stage and thus can already emerge during the prodromal phase of the disease. However, dysphagia is frequently overlooked in standard neurological assessments and is typically not diagnosed until later stages of the disease progression.^2^ Further, dysphagia in people with PD may impact all phases of swallowing^3^ involving compromised efficiency and safety of swallowing. This may contribute to malnutrition, dehydration, a diminished quality of life, and an increased risk of pneumonia, which is a leading cause of mortality in individuals with PD.^4^ In addition, dysphagia may impair the transport and intestinal absorption of dopaminergic medication, the mainstay treatment for PD, which has direct clinical implications for motor symptom management.^5^, ^6^ It is crucial to understand how swallowing function in people with PD differs from age‐related swallowing changes observed in healthy individuals for effective early intervention and treatment planning.

The assessment method used to evaluate swallowing function or swallowing‐related quality of life in PD can significantly influence the study's findings. Different assessment techniques provide varying levels of detail as well as sensitivity and specificity, which can ultimately affect how swallowing difficulties are identified and interpreted. Instrumental assessments, such as imaging techniques (eg, videofluoroscopy [VFS], flexible endoscopic evaluation of swallowing [FEES]) or pressure measures (eg, pharyngeal manometry), offer more precise, quantifiable data on swallowing biomechanics. These methods can highlight physiological abnormalities that may not be captured through clinical observation alone. In addition, functional methods (eg, surface or intramuscular electromyography [s/iEMG], electroencephalography [EEG], electropalatography [EPG]) can provide complementary insights into swallowing physiology and neuromuscular control. In contrast, patient‐reported outcome measures (PROMs) offer insight into the patient's perception of swallowing problems. PROMs assessing self‐perception of swallowing may not always correlate with more objective measures, as patients' perceptions of their condition can be influenced by a sensory mismatch. Only 20–40% of people with PD are aware of their swallowing issues, partly due to impaired laryngeal sensation,^7^, ^8^ which contributes to silent aspiration, and deficits in proprioception, a major feature of PD affecting most sensorimotor functions.^9^ Moreover, cognitive impairment, which is common in PD,^10^ may further affect the reliability and interpretation of PROMs, as it can influence self‐awareness and reporting accuracy. Thus, the choice of assessment method can influence the identification of swallowing impairments, the severity of dysphagia reported, and the overall management plan.

Recent comprehensive overviews of swallowing function and dysphagia in PD^2^, ^7^ often lack a systematic framework for selecting and reporting data, as is typically provided by a systematic or scoping review. Consequently, there is a need for a more structured overview of swallowing function in PD, encompassing various assessment methods used and the associated findings. This scoping review aimed to systematically identify reported swallowing characteristics and swallowing‐related quality of life in people with PD and the swallowing assessment methods. The research questions of this review were:

1. Which oropharyngeal and/or esophageal swallowing characteristics in people with PD are reported in the literature?
2. Which assessment methods are reported to evaluate swallowing in people with PD in the literature?

## 1 Materials and Methods

A scoping review was performed to meet the wide scope of the research aims.^11^ The review was conducted in accordance with the Joanna Briggs Institute's (JBI) methodology.^12^ The Preferred Reporting Items for Systematic reviews and Meta‐Analyses extension for Scoping Reviews (PRISMA‐ScR) were followed in reporting the review^12^ (Supplemental Material S1). A study protocol was registered and published in February 2024 on the Open Science Framework (https://osf.io/8b3hm). Deviations from the protocol, which emerged during the research process, are also described in the following sections.

### 1.1 Eligibility Criteria

This review included original quantitative and qualitative research articles that investigated swallowing function and swallowing‐related quality of life in adults (≥ 18 years) with PD. Articles published in the English or German languages were considered. The review encompassed studies from database inception up to February 15, 2024 to capture the full spectrum of research findings in this area.

Studies were excluded if they only described the presence or absence of penetration and/or aspiration without providing specific findings related to swallowing function. Additionally, studies were excluded if the primary focus was drooling, oral health, or coughing rather than swallowing. Data from participants with atypical parkinsonian syndromes, secondary parkinsonian disorders, or participants undergoing deep brain stimulation were also excluded. Reviews, summarizing empirical studies, validation studies, and studies focusing on interventions, prevalence, or the assessment of new diagnostic tools, were excluded.

### 1.2 Search Strategy

The databases searched included AMED, CINAHL, EMBASE, MEDLINE, Web of Science, and ProQuest Dissertations & Theses. The search strategy, including all identified keywords and index terms, was adapted for each database. ClinicalTrials.gov was searched for unpublished studies. In addition to database searches, the reference lists of all included studies were screened for further eligible articles. More detailed information on the search strategy is provided in the study protocol.^13^

### 1.3 Study Selection

All identified citations were collated and uploaded to the online platform Covidence (www.covidence.org) and duplicates were removed using the automated procedure within Covidence and manually verified for accuracy by two reviewers (J.Hi., K.E.). All titles and abstracts were screened based on the inclusion criteria by two independent reviewers (J.Hi., K.E.). The full texts of selected citations were assessed in detail against the eligibility criteria by the same two reviewers using a decision matrix, as described in the study protocol. If studies were unobtainable, authors were contacted. Studies were excluded if there was no reply from authors after a 2‐week period. Reasons for exclusion at full‐text stage were recorded. Any disagreements that arose between the reviewers at each stage of the selection process were resolved through discussion, or if needed with an additional reviewer (T.W.).

### 1.4 Data Extraction

Data were extracted to an Excel spreadsheet by three independent reviewers (J.Hi., K.E., K.W.) using a piloted data extraction tool, as per the study protocol. The data extracted included general study information, data about the studied population/participants, and key findings relevant to the review questions (swallowing characteristics and assessment methods). Data on swallowing characteristics were enhanced by incorporating information on bolus consistency following the International Dysphagia Diet Standardisation Initiative (IDDSI),^14^ bolus volume, single versus multiple swallows, as well as the significance and direction of the results (J.Ho., K.E.). For studies where no IDDSI level was reported, an estimated score range (eg, IDDSI levels 1–4 for trials with nectar‐ or pudding‐like consistencies) was assigned based on descriptions of consistencies provided in the studies (J.Ho., K.E.).

After the pilot extraction of the first 10 full texts, it was observed that the inclusion of participants with dysphagia in the studies was handled very differently, particularly with regard to the definition and assessment of dysphagia. This prompted the question of whether it was feasible to differentiate dysphagia in individuals with PD from general age‐related changes in swallowing in the absence of a control group. Consequently, the reviewers refined the inclusion criteria for this study as outlined in the section on Section 2.1 Eligibility Criteria, independently re‐screened the previously included full texts based on the revised criterion, and ultimately included only those studies in which swallowing function in individuals with PD was compared with that of a healthy control (HC) group. The extracted key data to answer the research questions were verified by two independent reviewers (K.E., K.W.).

Swallowing characteristics were extracted and defined as PD‐specific (a) if they were observed in the majority of PD participants (≥50%) and/or (b) if the group comparison of PD participants and HC revealed statistically significant results. Directions of swallowing characteristics in people with PD compared with HC were assigned using the following definitions: increased (greater in amplitude, quantity, or intensity), reduced (smaller in amplitude, quantity, or intensity), prolonged (extended duration), shortened (reduced in duration), delayed (later timing; onset or start of initiation happens later in time), and premature (occurring sooner than expected; initiation or onset happens earlier in time).

In some studies, the PD population was divided based on the presence or absence of dysphagia. For these studies, data from people with dysphagia compared with the HC group was included. When the PD group was categorized into younger and older individuals, data from the older group was included, as they were considered more age‐matched to the HC group.

### 1.5 Data Analysis

Data on swallowing characteristics were categorized into oropharyngeal‐ and esophageal‐stage difficulties.^15^ Subsequently, all characteristics were grouped into categories (eg, timing of oropharyngeal swallowing components, laryngeal movement) to group similar characteristics. The categorization was based on the nature of the characteristic, such as the type of measurement (eg, temporal, spatial, or physiological parameters) and the unit of measurement (eg, seconds, millimeters, or amplitude), ensuring a systematic and coherent classification for analysis. Similar characteristics were then compared to identify those that describe the same phenomenon, despite potential differences in the swallowing assessment tools (eg, masticatory duration measured by surface electromyography [sEMG] activity or via stopwatch in a clinical swallow evaluation [CSE]).

Swallowing characteristics were included in the final analysis if they were observed in the majority of PD participants (≥50%) and/or showed significant differences between participants with PD and HC and were described in at least two studies. This approach ensured the consistency and reliability of the results across diverse populations and research teams. For characteristics in the final analysis, results of non‐significant findings were added if they were reported in at least two studies.

The methods of swallowing assessment were categorized into instrumental assessment techniques, clinical assessments, and PROMs. Questionnaires that assessed only a single swallowing‐related question by rating the presence or absence of a swallowing problem were excluded from the analysis (eg, non‐motor symptoms questionnaire^3^).

## 2 Results

### 2.1 Search Results

**FIG. 1:**
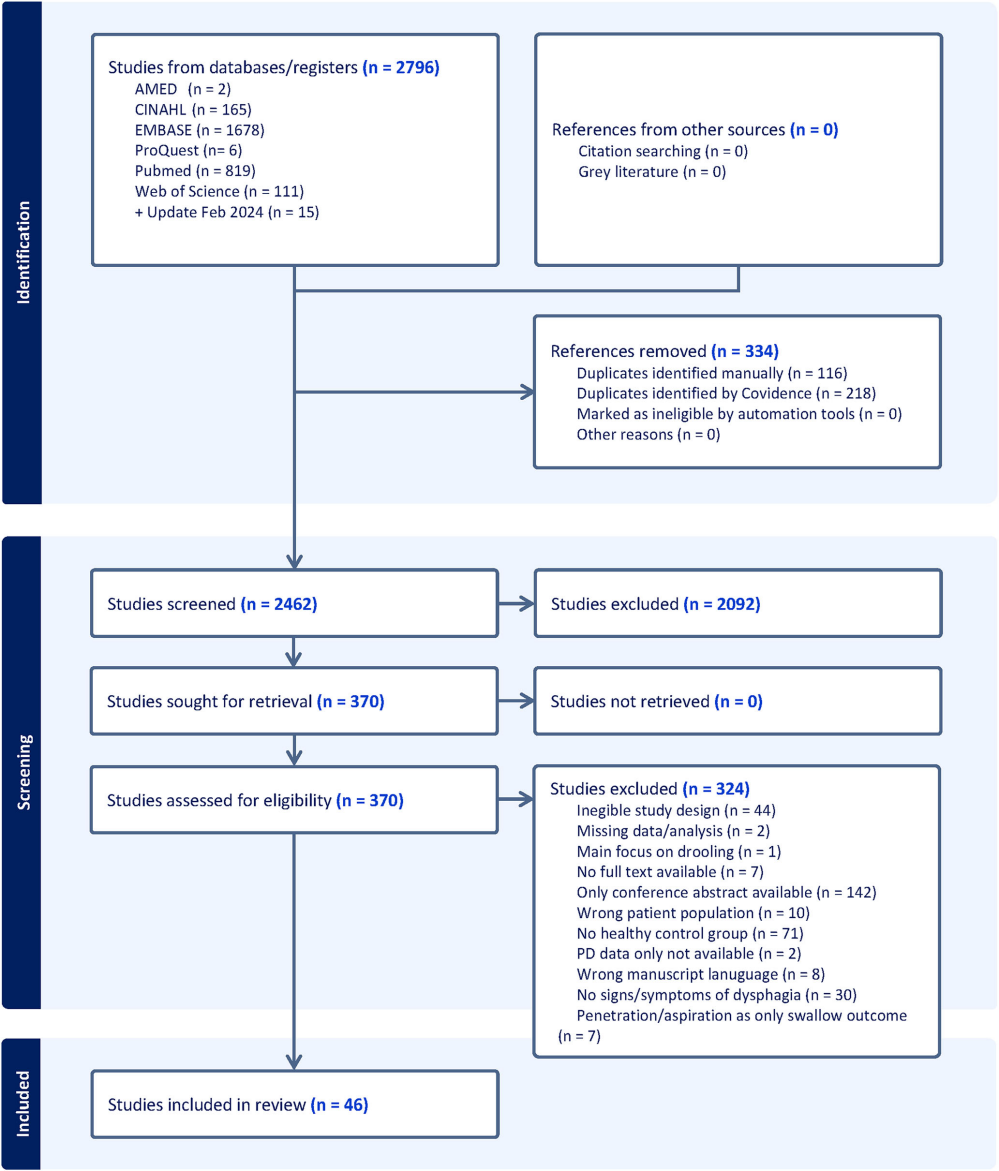
PRISMA‐ScR (Preferred Reporting Items for Systematic reviews and Meta‐Analyses extension for Scoping Reviews) flow diagram illustrating flow of information through the different phases of the scoping review. [Color figure can be viewed at wileyonlinelibrary.com]

The search identified 2796 study abstracts for screening. Some 334 duplicates were removed, 2462 abstracts were screened, and 2092 studies that did not meet the inclusion criteria were excluded following screening. A total of 370 studies were reviewed at full‐text stage for potential inclusion. Of these, 324 studies were excluded. Interrater agreement during title and abstract screening was 92% (Cohens's *κ* = 0.84) and during full‐text screening 95% (Cohens's *κ* = 0.90), which corresponds to strong and almost perfect agreement, respectively.^16^ Forty‐six studies were included in the scoping review (Fig. 1).

### 2.2 General Study Characteristics

Table 1 outlines the general characteristics of included studies. Most studies were conducted in the USA (9/46, 20%),^17^, ^18^, ^19^, ^20^, ^21^, ^22^, ^23^, ^24^, ^25^ Brazil (5/46, 11%),^26^, ^27^, ^28^, ^29^, ^30^ Germany (4/46, 9%),^3^, ^31^, ^32^, ^33^ and Republic of Korea (4/46, 9%).^34^, ^35^, ^36^, ^37^ A total of 1797 participants with PD aged between 35 and 85 years took part. Six studies did not report the sex of the included participants.^22^, ^29^, ^32^, ^38^, ^39^, ^40^ Therefore, the sex of 254 of the 1797 included participants remains unknown. In the remaining studies, the majority of participants identified as male (1005/1543, 65%). The severity of PD was rated predominantly using the Hoehn and Yahr (H&Y) scale^41^ (38/46, 83%).^3^, ^17^, ^18^, ^19^, ^20^, ^24^, ^26^, ^27^, ^28^, ^29^, ^31^, ^33^, ^35^, ^36^, ^37^, ^38^, ^40^, ^42^, ^43^, ^44^, ^45^, ^46^, ^47^, ^48^, ^49^, ^50^, ^51^, ^52^ Participants of all five H&Y stages were represented in the included studies. Most studies investigated swallowing characteristics in the oropharyngeal phase of swallowing (44/46, 96%)^3^, ^18^, ^19^, ^20^, ^21^, ^22^, ^23^, ^24^, ^25^, ^26^, ^27^, ^28^, ^29^, ^30^, ^31^, ^32^, ^33^, ^34^, ^35^, ^36^, ^37^, ^38^, ^39^, ^40^, ^42^, ^43^, ^44^, ^45^, ^46^, ^47^, ^48^, ^49^, ^50^, ^51^, ^52^, ^53^, ^54^, ^55^, ^56^, ^57^, ^58^, ^59^, ^60^, ^61^ and two studies assessed swallowing characteristics of the esophageal phase (2/46, 4%).^17^, ^62^ Five studies assessed PROMs considering the entire swallowing process (5/46, 11%).^18^, ^20^, ^37^, ^39^, ^40^

**Table 1:** General study information.

No.	Authors	Year of publication	Country	PD (n)	HC (n)	Male PD (n, (%))	PD age in years (mean (range))	H&Y stage	Swallowing phase^a^	Assessment
1	Alfonsi et al.	2007	Italy	28	24	20 (71)	66 (48–84)	N/S	1	iEMG, sEMG, LPM
2	Ali et al.	1996	Australia	19	23	N/S	73 (65–81)	1, 2, 3, 5	1	VFS, MAN
3	Ardenghi et al.	2021	Brazil	19	19	13 (68)	61.5 (54.4–68.6)	2–4	1	FEES
4	Baijens et al.	2011	The Netherlands	10	10	7 (70)	65.5 (50–80)	1–3	1	FEES, VFS
5	Bakke et al.	2011	UK	15	15	6 (71)	N/S (61–82)	2–4	1	NOT‐S, CSE^b^
6	Baram et al.	2023	Denmark	20	20	6 (40)	68.5 (35–80)	1–4	1	sEMG, TWST
7	Bassotti et al.	1998	USA	18	18	10 (56)	73 (57–86)	1–4	2	MAN, VQ
8	Belo et al.	2014	Brazil	10	10	6 (60)	66 (49–80)	1–3	1	sEMG
9	Buhmann et al.	2019	Germany	118	32	79 (67)	69 (59–79)	1–4	1	FEES
10	Clarke et al.	1998	USA	64	80	39 (61)	66.7 (50–83)	1–5	1	CSE^b^, TWST
11	Coriolano et al.	2012	Brazil	15	15	7 (47)	N/S (45–81)	1–3	1	sEMG
12	da Costa et al	2023	Brazil	23	24	N/S	64.9 (SD 13.7)	2.5 (1.5‐3)	1	VFS
13	Gandhi et al.	2022	Canada	20	20	16 (80)	70 (median) (65–75 IQR)	1–4	1	IOPI
14	Gandhi et al.	2023	Canada	17	78	13 (77)	69 (53–85)	2 (mean)	1	VFS
15	Gross et al.	2008	USA	25	25	25 (100)	71 (53–84)	2.4 (mean)	1	sEMG, nasal airflow, plethysmographic signals
16	Hammer et al.	2013	USA	18	18	9 (50)	73 (59–82)	2–4	1	FEES, SSQ
17	Kanna and Bhanu	2014	India	100	100	70 (70)	(50–70)	N/S	1	TWST
18	Kim et al.	2015	Republic of Korea	33	33	20 (61)	69.09 (±1.42)	1–5	1	VFS
19	Kim and Watts	2021	USA	14	10	6 (43)	72.5 (61–80)	2, 3	1	sEMG
20	Labeit et al.	2020	Germany	50	50	N/S	67.5 (± 8.39)	1–4	1	FEES
21	Lee et al.	2019	Republic of Korea	23	23	12 (52)	70.8 (± 6.6)	N/S	1	VFS
22	Leow et al.	2010	New Zealand	32	16	16 (50)	68.5 (45.8–82.5)	16 subjects ≤ stage 2; 16 subjects stage ≥ 2.5	1, 2	SWAL‐QOL
23	Marques et al.	2023	Brazil	20	10	13 (65)	68.7 (± 9.7)	2.7 (± 1.1) (mean)	1	HRM
24	Minagi et al.	2018	Japan	30	20	14 (47)	69.4 (55–81)	2–4	1	EPG
25	Nagaya et al	1998	Japan	16	15	7 (44)	70.2 (52–84)	3–5	1	VFS
26	Nascimento et al.	2020	Spain	50	12	26 (52)	70.46 (± 10.03)	1–4	1	VFS
27	Nilsson et al.	1996	Sweden	75	292	43 (57)	71 (43–85)	1–4	1	ROSS
28	Noble et al.	2015	UK	119	110	N/S	71.36 (45–91)	1–5	1	TWST, NVQ
29	Oh et al.	2021	Republic of Korea	30	50	15 (50)	21	1–4	1	VFS
30	Oommen et al.	2021	USA	11	61	N/S	74.45 (59–92)	N/S	1	IOPI
31	Pflug et al.	2018	Germany	119	32	80 (67)	68.9 (± 10.1)	1–5	1	FEES, TWST
32	Pflug et al.^c^	2019	Germany	115	32	76 (66)	68.6 (± 10.2)	1–5	1	FEES
33	Pinnington et al.	2000	UK	12	14	7 (58)	68	2–5	1	EDAT
34	Pitts et al.	2018	USA	28	28	17 (61)	71.1 (± 8.0)	N/S	1	IOPI
35	Potulska et al.	2003	Poland	18	22	6 (33)	69.3 (56–81)	1–3	1	sEMG
36	Robbins et al.^24^	1986	USA	6	6	5 (83)	69 (64–81)	1, 2, 3, 5	1	VFS
37	Schiffer and Kendall	2019	USA	68	48	49 (72)	71.7 (52–86)	N/S	1	VFS
38	Sulena et al.	2017	India	26	20	18 (69)	PD, MSA and PSP together 62.4 (±8.37)	N/S	1	TWST
39	Sung et al.	2010	Republic of Korea	54	26	22 (41)	67.1 (33–86)	1–3	1, 2	MAN, NVQ
40	Uludag et al.	2016	Turkey	21	18	13 (62)	68 (51–80)	1, 2, 4	1	EEG, sEMG
41	Umay et al.	2019	Turkey	120	60	42 (35)	63.3 (SD 8.67)	Mean 2.08 (SD 0.15)	1	sEMG
42	Wakasugi et al.	2017	Japan	201	20	106 (53)	70.6 (± 8.0)	1–5	1	VFS
43	Wang et al.	1994	Taiwan	27	27	21 (78)	65.93 (53–78)	N/S	2	Scintigraphy
44	Wang et al.	2017	Taiwan	42	37	27 (64)	64.7 (± 8.6)	1–3	1	sEMG
45	Wintzen et al.^61^	1994	The Netherlands	22	15	17 (77)	62.7(SD 9.9)	1–4	1	VFS
46	Wintzen et al.^62^	1994	The Netherlands	22	15	17 (77)	62.7 (SD 9.9)	1–4	1	VFS

Abbreviations: PD, Parkinson's disease; HC, healthy controls; H&Y, Hoehn & Yar; N/S, not specified; iEMG, intramuscular electromyography; sEMG, surface electromyography; LPM, laryngeal‐pharyngeal mechanogram; VFS, videofluoroscopic swallowing study; MAN, manometry; FEES, fiberoptic endoscopic evaluation of swallowing; NOT‐S, Nutritional and Oral Test for Swallowing; CSE, clinical swallowing evaluation; TWST, Timed Water Swallow Test; VQ, validated questionnaire; SD, standard deviation; IQR, interquartile range; SSQ, Swallowing Severity Questionnaire; SWAL‐QOL, Swallowing Quality of Life Questionnaire; HRM, high‐resolution manometry; EPG, electropalatography; ROSS, repetitive oral suction swallow; NVQ, non‐validated questionnaire; IOPI, Iowa Oral Performance Instrument; EDAT, Exeter Dysphagia Assessment Technique; MSA, multiple system atrophy; NMS, non‐motor symptoms questionnaire; PSP, progressive supranuclear palsy; EEG, electroencephalography.

^a^ Swallowing phase: 1 – oropharyngeal phase, 2 – esophageal phase.

^b^ CSE can consist of a patient interview and clinical swallowing assessment performed by the clinician. Therefore, it can include both patient‐reported outcome measures (PROMs) and investigator‐reported outcome measures (IROMs).

^c^ Participants of Pflug et al. 2018^3^ and 2019^33^ and Wintzen et al. 1994^60^ and 1994^61^ were only counted once for the analysis of the general study information, since it was supposedly the same participant cohort.

### 2.3 Characteristics of Swallowing Function in People with PD

A total of 317 swallowing characteristics were identified across the 46 studies. Of these, 153 characteristics were identified as significant in people with PD (found in the majority of PD participants [≥50%] or statistically significant compared with HC) and 164 were non‐significant compared with HC (Supplemental Material S2). Nineteen of the 153 oropharyngeal swallowing characteristics were identified as significant and reported in at least two independent studies (Table 2). Six of these 19 significant characteristics were identified as non‐significant in different studies. The swallowing characteristics studied most frequently were postswallow (oro‐)pharyngeal residue (13 studies),^3^, ^17^, ^20^, ^24^, ^26^, ^29^, ^31^, ^34^, ^35^, ^38^, ^45^, ^56^, ^60^ penetration and aspiration events (10 studies)^3^, ^20^, ^24^, ^29^, ^33^, ^34^, ^35^, ^38^, ^56^, ^60^ and swallowing speed/duration (9 studies).^18^, ^27^, ^28^, ^33^, ^40^, ^43^, ^48^, ^54^, ^57^ The results of all 19 swallowing characteristics were consistent in the direction of the result, except for vertical hyoid excursion, which yielded opposing results. No swallowing characteristics that were significant and independently investigated by at least two studies were identified for the esophageal phase of swallowing.

**Table 2:** Swallowing characteristics in people with Parkinson's disease compared with healthy controls.

	Swallowing characteristic	Study	Tool	Direction of result
Oral phase	Lingual movement (lingual tremor)	Ali 1996	VFS	Increased
		Robbins 1986^24^	VFS	
	Masticatory duration	Bakke 2011	CSE(stopwatch)	Prolonged
		Baram 2023	sEMG	
	Transit time – oral	Nilsson 1996	ROSS	Prolonged
		Pinnington 2000	EDAT	
		Oh 2021	VFS	Not sig.
		Wakasugi 2017	VFS	
	Piecemeal deglutition/swallow	Ali 1996	VFS	Increased
		Coriolano 2012	sEMG	
		Minagi 2018	EPG	
		Nagaya 1998	VFS	
		Robbins 1986^24^ ^,^ ^a^	VFS	
		Bajiens 2011	VFS	Not sig.
		Robbins 1986^24^ ^,^ ^a^	VFS	
		Sung 2010	PROM(NVQ)	
		Wintzen 1994^61^	VFS	
	Number of swallows to drink liquids	Baram 2023	sEMG	Increased
		Belo 2014	sEMG	
		Coriolano 2012	sEMG	
		Pinnington 2000	EDAT	
Oropharyngeal phase	Coughing before, during or after liquid intake	Clarke 1998	PROM(CSE)	Increased
		Hammer 2013	PROM(SSQ)	(quantity)
	Duration of suprahyoid/submental muscle activity	Alfonsi 2007	sEMG	Prolonged
		Kim 2021	sEMG	
	Swallowing capacity (mL/s)	Baram 2023	TWST	Reduced
		Nilsson 1996	ROSS	
	Swallowing speed/duration (e.g. s, s/swallow)	Baram 2023	sEMG, TWST	Prolonged
		Belo 2014	EMG, TWST	
		Clarke 1998	TWST	
		Coriolano 2012	sEMG, TWST	
		Kanna 2014	TWST	
		Nilsson 1996	ROSS	
		Noble 2015	TWST	
		Pflug 2019	FEES, TWST	
		Sulena 2017	TWST	
	Swallowing volume (mL/swallow)	Belo 2014	sEMG, TWST	Reduced
		Clarke 1998	TWST	
		Kanna 2014	TWST	
		Nilsson 1996	ROSS	
	Postswallow (oro‐) pharyngeal residue	Ali 1996	VFS	Increased
		Ardenghi 2021	FEES	
		da Costa 2023	VFS	
		Hammer 2013^a^	FEES	
		Kim 2015^a^	VFS	
		Lee 2019^a^	VFS	
		Nagaya 1998	VFS	
		Pflug 2018	FEES	
		Robbins 1986^24^	VFS	
		Bajiens 2011	VFS	Not sig.
		Bassotti 1998	PROM(VQ)	
		Hammer 2013 ^a^	FEES	
		Kim 2015 ^a^	VFS	
		Lee 2019^a^	VFS	
		Nagaya 1998	VFS	
		Robbins 1986^24^	VFS	
		Wintzen 1994^61^	VFS	
Pharyngeal phase	Initiation of pharyngeal swallow	Alfonsi 2007	sEMG	Delayed
		Robbins 1987	VFS	
		Bassotti 1998	PROM(VQ)	Not sig.
		Lee 2019	VFS	
		Ertekin	sEMG	
	Laryngeal elevation (total) for liquid (barium) intake	Marques 2023	VFS	Reduced
		Wintzen 1994^61^	VFS	
		da Costa 2023	VFS	Not sig.
		Lee 2019	VFS	
		Kim 2015	VFS	
	Hyoid excursion (horizontal movement – in mm)	Kim 2015^a^	VFS	Reduced
		Lee 2019	VFS	
		Marques 2023	VFS	
	Hyoid excursion (vertical movement – in mm)	Marques 2023	VFS	Reduced
		Kim 2015^a^	VFS	Increased
		Baijens 2011	VFS	Not sig.
		Lee 2019	VFS	
	Pharyngeal movement	Ali 1996	VFS	Reduced
		da Costa 2023	VFS	
	UES opening	Ali 1996	VFS	Reduced
		Kim 2015^a^	VFS	
	UES relaxation pressures (Nadir)	Marques 2023	HRM	Increased
	(Hypo)pharyngeal intrabolus pressure	Ali 1996	MAN	Increased
		Marques 2023	HRM	
	Penetration & aspiration events	da Costa 2023	VFS	Increased
		Kim 2015^a^	VFS	
		Lee 2019	VFS	
		Nagaya 1998	VFS	
		Pflug 2018^a^	FEES	
		Ali 1996	VFS	Not sig.
		Hammer 2013	VFS	
		Pflug 2018^a^	FEES	
		Robbins 1996^24^	VFS	
		Wintzen 1994^61^	VFS	

*Note*: Grey font indicates significant and non‐significant results within one swallowing characteristics.

Abbreviations: VFS, videofluoroscopic swallowing study; CSE, clinical swallowing evaluation; sEMG, surface electromyography; ROSS, repetitive oral suction swallow; EDAT, Exeter Dysphagia Assessment Technique; EPG, electropalatography; NS, not significant; PROM, patient‐reported outcome measure; NVQ, non‐validated questionnaire; SSQ, Swallowing Severity Questionnaire; TWST, Timed Water Swallow Test; FEES, flexible endoscopic evaluation of swallowing; VQ, validated questionnaire; UES, upper esophageal sphincter. HRM, high‐resolution manometry; MAN, manometry.

^a^ Studies found conflicting results regarding different consistencies or volumes.

### 2.4 Assessment Methods of Swallowing Function and Swallowing‐Related Quality of Life

A range of assessment tools were used to investigate swallowing in people with PD (Table 3). In most studies one assessment tool was used (29/46, 63%),^21^, ^22^, ^23^, ^24^, ^25^, ^26^, ^29^, ^31^, ^32^, ^33^, ^34^, ^35^, ^36^, ^39^, ^42^, ^46^, ^47^, ^48^, ^49^, ^50^, ^52^, ^54^, ^55^, ^56^, ^57^, ^58^, ^60^, ^61^, ^62^ while a smaller number of studies used multiple assessment tools (two or three) (17/46, 37%).^3^, ^17^, ^18^, ^19^, ^20^, ^27^, ^28^, ^30^, ^37^, ^38^, ^40^, ^43^, ^44^, ^45^, ^51^, ^53^, ^59^ Assessment methods included instrumental assessments (39/46, 85%),^3^, ^17^, ^19^, ^20^, ^21^, ^22^, ^23^, ^24^, ^25^, ^26^, ^27^, ^28^, ^29^, ^30^, ^31^, ^32^, ^33^, ^34^, ^35^, ^36^, ^37^, ^38^, ^42^, ^43^, ^45^, ^46^, ^47^, ^49^, ^50^, ^51^, ^52^, ^53^, ^55^, ^56^, ^58^, ^59^, ^60^, ^61^, ^62^ CSE at bedside (10/46; 22%),^3^, ^18^, ^27^, ^28^, ^40^, ^43^, ^44^, ^48^, ^54^, ^57^ and PROMs (7/46, 15%).^17^, ^18^, ^20^, ^37^, ^39^, ^40^, ^44^

**Table 3:** Assessment methods used and their number of reports across all studies.

Category		Total number of usages (n)	Assessment tools
Instrumental	Imaging	21	FEES, VFS
	Functional	18	EDAT, EEG, EPG, FSR sensors, iEMG, LPM, nasal airflow and plethysmographic signals, sEMG, scintigraphy
	Pressure	7	HRM, MAN, IOPI
Clinical		12	CSE, NOT‐S (examination), ROSS, TWST
PROM		7	CSE (interview), NOT‐S (interview), (N)VQ, SSQ, SWAL‐QOL

Abbreviations: FEES, flexible endoscopic evaluation of swallowing; VFS, videofluoroscopic swallowing study; EDAT, Exeter Dysphagia Assessment Technique; EEG, electroencephalography; EPG, electropalatography; FSR, Force Sensing Resistor; iEMG, intramuscular electromyography; LPM, laryngeal‐pharyngeal mechanogram; sEMG, surface electromyography; HRM, high‐resolution manometry; MAN, manometry; IOPI, Iowa Oral Performance Instrument; CSE, clinical swallowing evaluation; NOT‐S, Nutritional and Oral Test for Swallowing; ROSS, Repetitive Oral Suction Swallow; TWST, Timed Water Swallow Test; PROM, patient‐reported outcome measure; NVQ, non‐validated questionnaire; VQ, validated questionnaire; SSQ, Swallowing Severity Questionnaire; SWAL‐QOL, Swallowing Quality of Life Questionnaire.

#### 2.4.1 Instrumental Assessments

Most of the studies using instrumental assessment used swallowing imaging techniques (22/46, 48%)^3^, ^20^, ^24^, ^25^, ^26^, ^29^, ^30^, ^31^, ^32^, ^33^, ^34^, ^35^, ^36^, ^38^, ^42^, ^45^, ^47^, ^56^, ^58^, ^60^, ^61^ (Table 3). Of these, 15/46 (33%)^24^, ^25^, ^29^, ^30^, ^34^, ^35^, ^36^, ^38^, ^42^, ^45^, ^47^, ^56^, ^58^, ^60^, ^61^ studies used VFS and 7/46 (15%)^3^, ^20^, ^26^, ^31^, ^32^, ^33^, ^45^ used FEES. One of these studies combined VFS and FEES,^45^ and two studies combined VFS and pressure measures (manometry).^30^, ^38^ Pressures were assessed with intraoral pressure measurements (3/46, 7%),^22^, ^23^, ^46^ pharyngesophageal manometry (3/46, 7%)^17^, ^30^, ^37^ or esophageal manometry (1/46, 4%).^17^ A variety of functional assessments were used, for example, EMG (10/46, 22%)^19^, ^21^, ^27^, ^28^, ^43^, ^50^, ^51^, ^52^, ^53^, ^59^ or swallowing respiratory measurements (2/46, 4%).^19^, ^49^

#### 2.4.2 Patient‐Reported Outcome Measures

A minority of studies (7/46, 15%)^17^, ^18^, ^20^, ^37^, ^39^, ^40^, ^44^ used patient‐reported scales or interviews which captured subjective dysphagia characteristics, dysphagia severity, or swallowing‐related quality of life. Four of the seven studies (57%)^17^, ^20^, ^39^, ^44^ employed validated questionnaires. Three of these questionnaires primarily assessed changes in eating behavior and swallowing function: the Nutritional and Oral Test for Swallowing (NOT‐S),^44^ the Swallowing Severity Questionnaire (SSQ),^20^ and the self‐developed questionnaire by Ruth et al.^17^, ^63^ The remaining validated questionnaire, the Swallowing Quality of Life Questionnaire (SWAL‐QOL), assesses swallowing‐related quality of life.^39^ Two studies employed unvalidated questions to evaluate swallowing‐related changes in swallowing function.^37^, ^40^

#### 2.4.3 Other (Non‐)Validated Clinical Assessments

Timed water swallowing tests (TWSTs) were the most frequently used assessment for swallowing capacity, swallowing volume, or swallowing speed (8/46, 17%).^18^, ^27^, ^28^, ^40^, ^43^, ^48^, ^54^, ^57^ Clinical assessments were used in two studies (2/46, 4%).^18^, ^44^ One study^48^ used a repetitive oral suction swallow test (ROSS)^64^, ^65^ to assess the functional swallowing capacity, suction strength, and coordination involved in oral motor function. One study^44^ used a manual muscle test within the CSE to assess muscle strength of the muscles involved in mastication.

## 3 Discussion

This scoping review identified 19 key oropharyngeal swallowing characteristics in people with PD compared with HC, which are supported by scientific evidence from independent studies. These 19 significant characteristics, drawn from 317 identified features, highlight both variability in the assessment of swallowing characteristics and limited replication across studies. Frequently examined features, such as postswallow residue, penetration–aspiration, and swallowing speed, appear to be key markers of dysphagia in PD. However, only prolonged swallowing speed/duration was consistently reported as significantly different across studies. Conflicting results for other characteristics and the paucity of data on the esophageal phase suggest that crucial aspects of swallowing impairment in PD remain insufficiently detected or quantified. Future research should prioritize replication and the use of standardized assessment methods to improve robustness, comparability, and sensitivity in identifying swallowing changes in PD.

### 3.1 Characteristics of Swallowing Function in People with PD

#### 3.1.1 Oral Phase

Swallowing characteristics of the oral stage include increased lingual movements (tremor),^24^, ^38^ prolonged mastication^43^, ^44^ and oral transit times (OTT),^48^, ^49^ and the need for multiple swallows to achieve bolus clearance of food and liquids.^24^, ^28^, ^38^, ^55^, ^56^ Lingual tremor is notably more pronounced in individuals with PD compared with HC.^24^, ^38^ However, the distinction between tremors observed during voluntary swallowing and at rest, which is most commonly observed in PD,^66^, ^67^ is critical. Resting tremor in PD typically diminishes once voluntary movement is initiated.^67^ Interestingly, Robbins et al.^24^ observed lingual tremor during the voluntary oral transit phase of swallowing, suggesting that the neural circuits responsible for tremor generation may influence bulbar and limb motor pathways differently. In contrast, Ali et al.^38^ did not specify the context of observation. More recent work focusing exclusively on individuals with PD by Dumican et al.^68^ demonstrated that an oropharyngeal resting tremor, including the tongue, can significantly affect swallowing (eg, alter timing, increase pharyngeal residue, and raise the risk of airway invasion), particularly with thin and high‐volume boluses. Therefore, specifying and differentiating between tremor types is important for future research to better understand the underlying neural mechanisms and their impact on swallowing function.

Prolonged masticatory duration^43^, ^44^ has been consistently observed in individuals with PD, likely related to bradykinesia and associated extrapyramidal symptoms. In contrast, findings regarding OTT are less consistent. While two studies using clinical assessments reported prolonged OTT in PD,^48^, ^49^ two other studies using VFS did not find significant differences compared with HC.^36^, ^58^ This may be due to VFS providing a more nuanced assessment of oral phase function. Overall, although prolonged masticatory duration appears to be a robust finding, evidence for prolonged OTT remains mixed, underscoring the need for cautious interpretation when discussing oral phase impairments in PD.

Lastly, piecemeal deglutition, where one bolus is swallowed in multiple attempts, and an increased number of swallows is required to drink liquids^24^, ^28^, ^38^, ^55^, ^56^ may also be due to motor impairments like bradykinesia and rigidity, affecting the coordination of swallowing muscles. Additionally, commonly observed sensory and proprioceptive deficits in PD may contribute to these difficulties.^20^ It is noteworthy that four of the nine studies investigating piecemeal deglutition did not yield significant results, despite utilizing largely the same instrumental assessment (VFS) and consistent definitions of piecemeal deglutition. This discrepancy may stem from variations in participant characteristics, such as age, underlying conditions, or swallowing function. These differences highlight the need for further research to explore how participant characteristics influence the detection and clinical relevance of this swallowing phenomenon.

#### 3.1.2 Oropharyngeal Phase

Compared with HC, individuals with PD exhibit reduced swallowing capacity (mL/s) and volume (mL/swallow), as well as prolonged swallowing speed (s, s/swallow).^18^, ^27^, ^28^, ^33^, ^40^, ^43^, ^48^, ^54^, ^57^ These characteristics are often interpreted as a result from bradykinesia, hypokinesia, and akinesia, leading to impaired bolus formation and propulsion.^69^, ^70^ However, intentionally limiting bolus size or swallowing volume could also serve as a strategy to enhance control and reduce the risk of airway invasion. In this sense, what appears as reduced efficiency may, in fact, represent an adaptive mechanism to preserve safety in the context of progressive motor impairment.

Postswallow (oro‐)pharyngeal residue, particularly in the valleculae, is commonly reported in individuals with dysphagia due to PD and is considered a marker of impaired swallowing efficiency.^7^, ^71^ While several studies using imaging techniques have identified postswallow residue as a significant finding,^3^, ^20^, ^24^, ^26^, ^29^, ^34^, ^35^, ^38^, ^56^ an equally notable number of studies using the same methods did not.^20^, ^24^, ^34^, ^35^, ^45^, ^56^, ^61^ This discrepancy highlights the complexity of assessing swallowing efficiency and the need for further investigation to understand the factors contributing to inconsistent findings in imaging‐based research. The presence or absence of postswallow residue may be influenced by both the severity of the swallowing impairment and the specific imaging modality and residue rating used.

#### 3.1.3 Pharyngeal Phase

Reduced pharyngo‐laryngeal movements, including total laryngeal elevation,^30^, ^60^ hyoid excursion (both horizontal and vertical),^30^, ^34^, ^35^ and the range of pharyngeal motion^29^, ^38^ are often attributed to rigidity, bradykinesia, or hypokinesia associated with PD. The restricted movement of the larynx and hyoid bone can result in impaired bolus propulsion, and increased risk of aspiration.^34^, ^72^

However, the findings regarding vertical hyoid excursion in people with PD are conflicting. One study^30^ suggests that vertical hyoid excursion is reduced in this population, which aligns with the general understanding of impaired muscle function in PD, due to, for example, hypokinesia. Another study^34^ did not find significant reductions in vertical hyoid excursion. Two studies,^35^, ^45^ also investigating vertical hyoid excursion, did not find significant results using the same instrumental assessment method. These discrepancies may be explained by several factors, such as differences in study design, patient characteristics, or the specific methods used to measure hyoid motion. Furthermore, the influence of other factors such as disease stage, medication effects, and individual variability in motor symptoms could also contribute to the inconsistencies. Overall, while reduced pharyngo‐laryngeal movement is a hallmark of PD‐related dysphagia, further research is needed to clarify the role of vertical hyoid excursion and use assessment methods that will increase our understanding of the nature of these impairments in swallowing function.

Participants with significantly decreased anterior hyoid bone movement also showed a poor upper esophageal sphincter (UES) opening.^34^ Two other studies also confirmed UES impairments in individuals with PD.^30^, ^38^ In detail, UES opening was reduced on VFS^38^ and the UES nadir relaxation pressures were increased using high‐resolution manometry compared with HC.^30^ An incomplete UES relaxation and a reduced UES opening, both associated with increased hypopharyngeal intrabolus pressure, were also found to be significantly prevalent in PD.^30^, ^38^

Given the wide range and complexity of oropharyngeal swallowing impairments, it is not surprising that penetration and aspiration events are reported to occur more frequently in individuals with these conditions.^3^, ^29^, ^34^, ^35^, ^56^ However, a considerable number of studies have also found non‐significant results,^3^, ^20^, ^24^, ^38^, ^61^ indicating that the relationship between swallowing impairments and the occurrence of penetration and aspiration may not be universally consistent. Notably, one study^3^ found conflicting results regarding different consistencies or volumes of swallowed material, suggesting that the severity and frequency of penetration and aspiration events may be influenced by these factors.

#### 3.1.4 Esophageal Phase

Only a few studies investigated swallowing characteristics in the esophageal phase of swallowing. Most of the results for this stage stem from PROMs asking for issues with swallowing food^18^, ^20^, ^37^, ^40^ or changes in swallowing‐related quality of life incorporating all stages of swallowing function.^39^ Two studies investigated the esophageal phase of swallowing using instrumental assessment (scintigraphy, manometry).^17^, ^62^ However, no significant and independently investigated swallowing characteristics were identified for the esophageal phase of swallowing. More research is needed in this underinvestigated phase of swallowing.

### 3.2 Assessment Methods Used to Evaluate Swallowing Function in People with PD

Across studies, VFS and FEES were the most frequently used instrumental assessments. These methods are typically used as the sole instrumental assessment and only one study combined the two methods.^45^ Although VFS and FEES are considered gold standards, they remain semi‐quantitative methods and depend on examiner‐based identification of anatomical landmarks. The choice of assessment tool may introduce bias in the identification of swallowing characteristics overall, as gold standard instruments are not only more frequently used but also limited to assessing only distinct biomechanical aspects of swallowing. A multimodal approach, integrating pressure‐based, functional, or clinical assessments, appears essential to achieve a more comprehensive characterization of swallowing physiology in PD.^7^

While VFS and FEES provide valuable insights into altered timing and reduced range of oropharyngeal motion, these methods only allow indirect inferences regarding the underlying pathophysiological mechanisms and do not directly reveal the neuronal or biochemical substrates involved. In PD, progressive degeneration of dopaminergic neurons in the substantia nigra leads to hallmark motor features including rigidity, tremor, and bradykinesia.^73^ While these features share a common etiological origin, recent studies indicate that their underlying mechanisms diverge at the level of basal ganglia circuitry and cortical involvement.^74^ Specifically, bradykinesia is primarily associated with dysfunction of the cortico‐basal ganglia‐thalamo‐cortical loop, leading to impaired initiation and scaling of voluntary movements. In contrast, rigidity appears to reflect increased background muscle tone, likely mediated by aberrant basal ganglia output in combination with altered spinal reflex modulation.^74^ A more comprehensive understanding of the cellular and systems‐level mechanisms contributing to these symptoms necessitates the integration of complementary methodologies, including advanced neuroimaging and electrophysiological techniques. Ultimately, while instrumental assessment can guide clinical management and rehabilitation strategies, addressing the core pathophysiology in future research is crucial for developing targeted therapies that slow or halt disease progression.

Healthcare professionals and researchers should consider that the results from clinical and instrumental swallowing assessments may not fully reflect an individual's actual eating and drinking abilities in everyday situations. Factors like environmental distractions, food and liquid consistency, bolus size, medication effects, motor fluctuations, and dyskinesias can influence test outcomes.^26^, ^75^ Therefore, it is crucial to observe people with PD during their typical eating and drinking routines or, alternatively, collect this information through a comprehensive case history or validated questionnaires.

### 3.3 Defining Parkinsonian Motor Deficits in Swallowing

The three classical motor deficits of PD—bradykinesia, hypokinesia, and akinesia—are reflected in the identified swallowing characteristics. Based on the findings in this review, oropharyngeal bradykinesia could be defined as a prolonged duration of relevant motility parameters compared with HC, such as the duration of suprahyoid or submental muscle activity or overall swallowing speed, primarily measured via sEMG or TWST.^18^, ^21^, ^27^, ^40^, ^43^, ^53^, ^54^, ^57^ However, evidence derived from gold standard assessment methods in combination with HC groups remain limited, providing initial indications of bradykinetic swallowing in PD (eg,^31^, ^33^, ^34^). Hypokinesia may underlie swallowing characteristics that reflect a reduced range of motion compared with HC, as evidenced by decreased laryngeal elevation or hyoid excursion, typically assessed through VFS. In studies using FEES, hypokinetic patterns may be inferred indirectly, for instance through the progressive accumulation of residue in the valleculae, described as a “build‐up phenomenon”.^3^ Finally, akinesia refers to the absence or significant delay in movement initiation, such as the delayed initiation of the pharyngeal swallow in individuals with PD. However, delayed swallowing may also be the result of a combination of motor impairments (akinesia, bradykinesia) and sensory deficits, particularly reduced pharyngeal and laryngeal sensitivity, which impairs timely triggering of the swallow response, according to the definition of Labeit et al.^32^

### 3.4 Strengths and Limitations

To the best of our knowledge, this is the first review to systematically synthesize and compare swallowing characteristics in people with PD. However, certain limitations should be considered. The results of the studies included individuals with PD with and without diagnosed dysphagia. In some studies, it was reported that dysphagia was diagnosed by a healthcare professional while other studies asked the person with PD (self‐reported dysphagia). In most cases, a specific description of the swallowing function or a criterion of dysphagia at inclusion in the study was not provided by the authors. Furthermore, studies exclusively investigating individuals with PD have been purposefully excluded from this review, which may skew the understanding of PD‐related dysphagia presented in this review when compared with other conditions.

In the general population, slightly more men than women are affected by PD, with studies showing a prevalence of 53.1% in males.^76^ However, in the included studies in this scoping review, 65% of participants with PD were male, indicating that women are underrepresented in this research area. This imbalance may affect the generalizability of our findings, as PD, and therefore dysphagia, may present differently in women.^77^ Furthermore, ethnicity was reported in only 4% of the included studies,^46^, ^47^ thereby neglecting the potential influence of cultural and ethnic factors on variations in symptom perception, therapeutic approaches, and healthcare accessibility. The gender imbalance and the lack of data on ethnicity was also observed in a review on intervention studies by Hirschwald et al.,^78^ further underscoring the need to address this gap in future research.

In addition, the included studies did not systematically control for age‐related changes or the presence of comorbidities, which may confound the interpretation of swallowing characteristics. Furthermore, potential fluctuations in motor performance due to dopaminergic treatment (ON and OFF states) were not consistently considered. As PD symptoms can vary substantially depending on medication state, this omission may significantly influence observed swallowing performance and its interpretation.

It should also be noted that the swallowing characteristics identified in the included studies do not appear to be specific to particular disease stages or dysphagia severity levels. This limits conclusions about their diagnostic value in clinical stratification or disease monitoring. While describing characteristic features may support early detection efforts (eg, in prodromal stages), care must be taken not to overinterpret these features as pathognomonic, as they may also occur in other conditions or patient groups. Comparative studies with other neurological and non‐neurological populations would be helpful to clarify specificity.

Different measurement methods, assessment tools, or variations in bolus consistencies, volumes, and instructions were partly used to investigate the characteristics, which may have led to differing results. Efforts were made to account for results obtained from different volumes and to compare only those results that were similarly defined or investigated. A few studies have investigated different variables in great detail, putting significant effort into their analysis.^79^, ^80^ Although this level of detail could not be incorporated into the data analysis of this review, the thorough approach of these studies is commendable.

## 4 Conclusions

This scoping review identified 19 oropharyngeal swallowing characteristics in people with PD that differ from those observed in HC. Although these characteristics are commonly investigated and reported in isolation, clinical experience and patient presentations indicate that multiple features often co‐occur within the same individual. This underscores the multifactorial nature of dysphagia in PD and suggests it should be conceptualized as a complex syndrome with diverse and interrelated manifestations.

Based on the findings of this review, a first definition of PD‐related dysphagia can be proposed as a complex swallowing movement disorder characterized by bradykinesia, hypokinesia, and akinesia within the swallowing mechanism. These manifest as prolonged swallowing durations, reduced movement amplitudes, and/or delayed pharyngeal swallow initiation, respectively.

It is important to acknowledge that based on these findings our understanding may be biased, as the choice of which characteristics are assessed is based on the researchers' decision. Additional swallowing characteristics may exist in this population but remain unnoticed or understudied. A combination of objective and subjective measures is often recommended to provide a comprehensive understanding of swallowing function in PD, allowing for more accurate diagnosis, individualized treatment planning, and monitoring of disease progression. Future research should aim to use more comprehensive assessment protocols to capture overlooked swallowing characteristics in PD and to clarify conflicting findings. This would improve our understanding of dysphagia in this population and inform more targeted therapeutic interventions.

## Author Roles

(1) Research Project: A. Conception, B. Design, C. Coordinated the Review Process, D. Conducted Literature Searches, E. Screened and Selected Studies, F. Performed Data Extraction, Analysis, and Synthesis, G. Contributed to Data Extraction and Interpretation, H. Provided Subject‐Matter Expertise in Dysphagia and Parkinson's Disease, I. Provided Methodological Expertise, J. Supervised Data Synthesis; (2) Manuscript Preparation: A. Writing of the First Draft, B. Revised the Manuscript, C. Editing of the Final Draft, D. Critically Revised the Manuscript for Intellectual Content, E. Reviewed and Approved the Final Version.

K.E.: 1A, 1B, 1C, 1E, 1F, 1G, 1H, 2A, 2B, 2C, 2E.

J.Hi.: 1A, 1B, 1D, 1E, 1G, 1H, 1I, 2C, 2E.

J.Ho.: 1G, 1H, 2C, 2E.

K.W.: 1G, 1H, 2C, 2E.

J.K.: 1A, 1B, 2C, 2E.

R.D.: 1H, 1I, 2C. 2E.

T.W.: 1A, 1B, 1H, 1J, 2C, 2D, 2E.

## Financial Disclosures and Conflicts of Interest

K.E. received a salary from the University of Applied Sciences and the Klinikum Osnabrueck that cover travel expenses to present the results of this review at conferences. Her position at the Osnabrück University of Applied Sciences was financed by funds from both the Federal Ministry of Research, Technology and Space (BMFTR) and the Lower Saxony Ministry for Science and Culture (MWK) as part of the “Professorinnenprogramm III” funding program granted to Osnabrück University of Applied Sciences. The funding bodies did not have any role in the study design or the writing of this protocol and did not have any role in the data collection and analysis, interpretation of data, or decision to publish the results of the study. J.Hi.: None. J.Ho. received a doctoral fellowship from the German Academic Scholarship Foundation (Studienstiftung des deutschen Volkes) and a 3‐month part‐time salary from the University of Applied Sciences Osnabrueck to support data extraction for this review. The funding bodies did not have any role in the study design or the writing of this protocol and did not have any role in the data collection and analysis, interpretation of data, or decision to publish the results of the study. K.W. received payment of travel and accommodation for speaking at the annual meeting of the Deutsche Gesellschaft für Ultraschall in der Medizin (Pädiatrie). K.W. is involved in research projects that receive partial financial support by the Deutschschweizer Logopädinnen und Logopädenverband DLV, the Förderstiftung für das Sprachheilwesen im Kanton Zürich, and the Sprachheilinstitutionen Schweiz through institutional support to the Swiss University of Speech and Language Sciences (hlo). No direct personal funding was received from the funders. For working on the development of a Swiss guideline for dysphagia management in Parkinson's disease, K.W. received payment by the Föderation der Schweizer Logopädinnen und Logopäden (FSLO). The guideline project is supported by the FSLO, Parkinson Schweiz, and the Eidgenössische Qualitätskommission (EQK). None of the funding bodies mentioned played a role in the development of the work presented in this manuscript. J.K.: None. R.D. has received honoraria for adviced services from Danone, he also has received honoraria for educational presentations from Argenx. T.W. received speaker fees/honoraria from Bial, AbbVie, Merz, Desitin, Pfizer, Britannia, Zambon, Neuraxpharm, Esteve, and Licher. He received payment of travel, accommodation, subsistence, and conference registration from AbbVie. He received payment for expert advice from Stadapharm, Bial, Merz, AbbVie, and Phagenesis. He received funding for research from AbbVie and Ever Pharma. This research did not receive any specific grant from funding agencies in the public, commercial, or not‐for‐profit sectors.

## Supporting information


**Data S1.** Supporting Information.

**Data S2.** Supporting Information.

## Data Availability

The data that supports the findings of this study are available in the supplementary material of this article.
